# Atomic structures and interfacial engineering of ultrathin indium intercalated between graphene and a SiC substrate†

**DOI:** 10.1039/d3na00630a

**Published:** 2023-09-08

**Authors:** Van Dong Pham, Chengye Dong, Joshua A. Robinson

**Affiliations:** a Paul-Drude-Institut für Festkörperelektronik, Leibniz-Institut im Forschungsverbund Berlin e.V. Hausvogteiplatz 5-7 10117 Berlin Germany pham@pdi-berlin.de; b Department of Materials Science and Engineering, The Pennsylvania State University, University Park PA USA; c Center for Nanoscale Science, The Pennsylvania State University, University Park PA USA; d Department of Physics, The Pennsylvania State University, University Park PA USA; e 2-Dimensional Crystal Consortium, The Pennsylvania State University, University Park PA USA; f Center for 2-Dimensional and Layered Materials, The Pennsylvania State University, University Park PA USA; g Materials Research Institute, The Pennsylvania State University, University Park PA USA; h Department of Chemistry, The Pennsylvania State University, University Park PA USA; i Center for Atomically Thin Multifunctional Coatings, The Pennsylvania State University, University Park PA USA

## Abstract

Two-dimensional metals stabilized at the interface between graphene and SiC are attracting considerable interest thanks to their intriguing physical properties, providing promising material platforms for quantum technologies. However, the nanoscale picture of the ultrathin metals within the interface that represents their ultimate two-dimensional limit has not been well captured. In this work, we explore the atomic structures and electronic properties of atomically thin indium intercalated at the epitaxial graphene/SiC interface by means of cryogenic scanning tunneling microscopy. Two types of surfaces with distinctive crystalline characteristics are found: (i) a triangular indium arrangement epitaxially matching the (√3 × √3)R30° cell of the SiC substrate and (ii) a featureless surface of more complex atomic structures. Local tunneling spectroscopy reveals a varying n-type doping in the graphene capping layer induced by the intercalated indium and an occupied electronic state at ∼−1.1 eV that is attributed to the electronic structure of the newly formed interface. Tip-induced surface manipulation is used to alter the interfacial landscape; indium atoms are locally de-intercalated below graphene. This enables the quantitative measurement of the intercalation thickness revealing mono and bi-atomic layer indium within the interface and offers the capability to tune the number of metal layers such that a monolayer is converted irreversibly to a bilayer indium. Our findings demonstrate a scanning probe-based method for in-depth investigation of ultrathin metal at the atomic level, holding importance from both fundamental and technical viewpoints.

## Abstract 2

The STM tip is used to visualize and to engineer the intercalated indium at the interface between graphene and SiC substrate with atomic precision.

## Graphical abstract

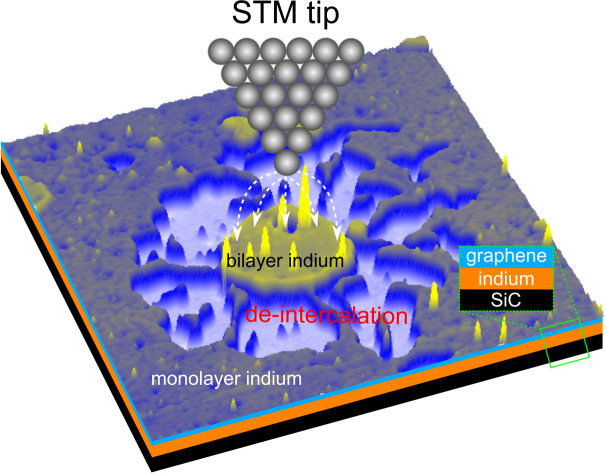

## 1. Introduction

Crystalline, atomically thin metals stabilized at the interface between epitaxial graphene (EG) and a silicon carbide (SiC) substrate exhibit remarkable physical properties owing to their distinct dimensionality and dominant quantum size effects.^1,2^ Depending on the intercalated metal element, intercalation thickness, atomic arrangement and interfacial bonding characters, various new physics might emerge in their two-dimensional (2D) forms such as superconductivity in Ga^3^ and Ca,^4^ semiconducting behaviors in Au,^5^ Ag,^6^ Ga and In^7^ and large nonlinear optical responses in 2D Ga and In^7^ which do not occur in their bulk counterparts. Together with 2D van der Waals (vdW) materials^8,9^ 2D metals are considered as compelling material platforms for quantum and optoelectronic technologies^10,11^ as well as for the investigation of new physical effects.^12^

The high energy of the EG/SiC interface provides a strong thermodynamic driving force^3^ for metal atoms to spontaneously squeeze themselves under graphene and form large-scale ordered ultrathin crystals typically in a range between one and a few atomic layers.^3,7^ A graphene top layer stabilizes the 2D metals at the interface and protects them effectively against environmental degradation. Sandwiched between EG and the SiC substrate, ultrathin metal serves as part of the vdW heterostructure which is immediately available for advanced experimental investigations and future applications.^3,13^ Upon intercalation, metal at the EG/SiC interface tends to match epitaxially with the uppermost atomic layers of the SiC substrate, forming a highly ordered triangular lattice as demonstrated for Ga,^3,7^ Au,^5,14^ Ag,^6^ Sn,^15,16^ and Cu^17^ through a wide range of investigation techniques including low-energy electron diffraction (LEED), low-energy electron microscopy (LEEM), angle-resolved photoemission spectroscopy (ARPES), cross-sectional scanning transmission electron microscopy (STEM), first principles density functional theory (DFT) and scanning tunneling microscopy (STM). In order to understand and exploit the rich physics of these interfacial 2D metals, it is significantly important to understand their microscopic details as well as their interaction within the EG/SiC interface at the atomic level.

Here, we reveal the atomic and electronic structures, the intercalation thickness and the interfacial engineering of the 2D indium using a cryogenic STM system. Indium is a group 13 metal in which its 2D form has not been well studied, nevertheless exhibiting various intriguing physics. For example, triangular monolayer indium on SiC (0001) displays non-trivial topological band inversion induced by spin–orbit coupling;^18^ (√7 × √3) In on Si (111) exhibits a nearly free electron 2D electron gas (2DEG) system^19^ or superconductivity behaviors;^20^ and ultrathin In confined at the EG/SiC interface shows an extraordinary nonlinear optical response.^7^ First, bias-dependent imaging is used to identify the atomic structure of In below graphene which is only sensitive to a specific bias voltage range. A differential tunneling spectrum (d*I*/d*V*) is used to probe the electronic structures of the EG/In/SiC interface revealing the electronic doping of graphene induced by the charge transfer from the intercalated metal and an occupied state arising from the newly formed interface. Second, tip-induced surface manipulation using a relatively large bias voltage pulse is applied between the sample and the tip to enable de-intercalation of the In atoms below graphene, thus facilitating an accurate measurement of the metal thicknesses inside the EG/SiC interface, and to further tune the atomic layer numbers of the In. Our results demonstrate microscopic evidence on the structural and electronic properties of the confined metal and feasibility to control the interfacial landscape with atomic precision using solely the STM tip.

## 2. Experimental details

### 2.1 Sample preparation

EG is first formed from SiC *via* silicon sublimation at 1800 °C, following our previous works.^3^ Here, we utilize partial-buffer EG, where we grow sub-monolayer EG (∼90% coverage), yielding regions where the graphene buffer layer (GBL) is exposed, to enable intercalation of In without the need for the oxygen-plasma treatment. The GBL consists of a graphene-like honeycomb structure in which one third of the carbon atoms in this layer are covalently bound to the Si atoms of the SiC substrate.^21^ Thus, this layer does not display the typical Dirac cone in its band structure as for graphene. This sample preparation provides a route to reduce surface roughness following intercalation, thereby enabling STM measurements of the surface following In intercalation. Atomically thin In is achieved *via* the confinement heteroepitaxy (CHet) in a horizontal tube furnace fitted with a 1 inch outer diameter quartz tube. Indium pellets are placed in an alumina crucible ∼5 mm below an EG/SiC substrate, and CHet is performed at 800 °C in a 500 Torr Ar environment (Ar flow of 50 sccm) for 30 minutes.

### 2.2 STM measurements

After In intercalation, the sample was transported under ambient conditions and transferred into the preparation chamber of the STM and degassed at 150 °C to remove the moisture from the surface. The sample was then transferred to the STM stage. All the STM experiments were performed in a cryogenic STM (Createc) operating under UHV (base pressure < 1 × 10^−10^ mbar) at a sample temperature of ∼5 K. Differential conductance d*I*/d*V* (*I*: tunneling current; *V*: sample bias voltage) was measured by using a lock-in technique amplifier with a peak-to-peak bias modulation of 5–10 mV and a modulation frequency of 675 Hz. Prior to all measurements, the STM tip is carefully calibrated by performing conductance spectra in the field emission regime (FER) on graphene/SiC using a high positive bias voltage (ranging from 2 V to 10 V) applied between the tip and the sample (see ESI, Fig. S1†).

## 3. Results and discussion

### 3.1 Atomic structures of the intercalated indium

**Fig. 1:**
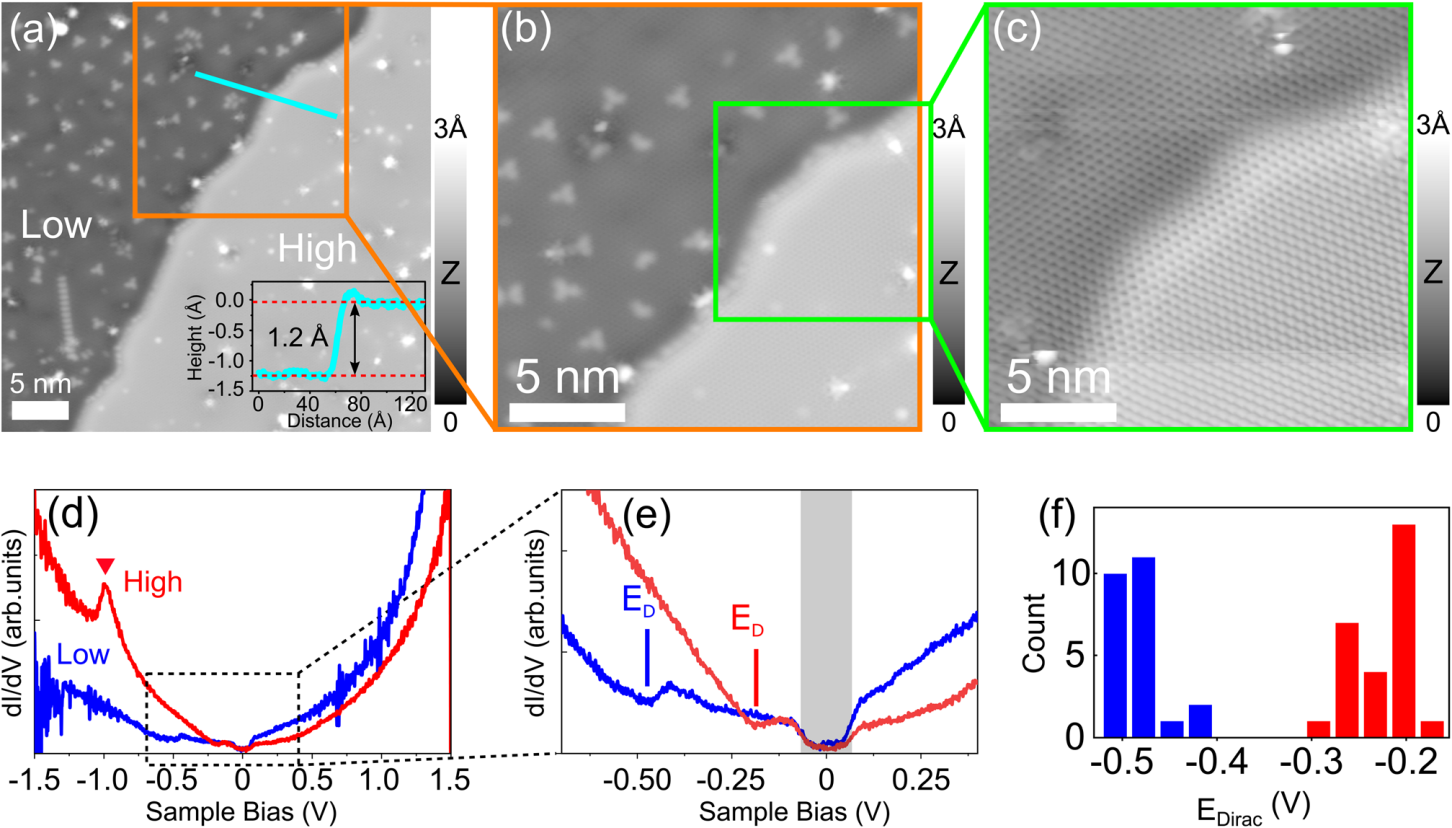
Topography and electronic structures of 2D In at the EG/SiC interface. (a) Representative STM topography image (*V* = 1 V, *I* = 10 pA) of In intercalated at the EG/SiC interface showing dark (indicated by “low”) and bright (“high”) regions formed by different In coverages under the graphene top layer. Inset: height profile over the boundary step between the two regions along the cyan line. (b) STM topography image (*V* = 1 V, *I* = 10 pA) of a zoomed-in area marked by an orange square in (a). (c) STM topography image (*V* = 0.1 V, *I* = 100 pA) of the green square area in (b) reveals the continuous honeycomb lattice of graphene over the boundary step between the low and high regions. (d) Representative d*I*/d*V* spectra obtained on clean areas in the low and high regions. A pronounced occupied state (indicated by the red triangle) is only observed in the high intercalated region while being absent on the lower one. (e) Zoomed-in d*I*/d*V* spectra indicated by the dashed rectangle in (d) shows a clear shift of the Dirac point in each region. (f) Histogram of Dirac point energy measured for graphene in the low (blue bars) and high (red bars) regions through different locations.

Indium is intercalated into the EG/SiC interface with two atomically thin thicknesses, resulting in low and high surface regions as shown in the representative STM image obtained at 1 V in Fig. 1(a). As can be seen, the surface displays two different intercalated regions referred to as “low” and “high”, respectively. At this bias voltage, each region appears as an atomically flat terrace with a surface corrugation below 0.1 Å (considered within one terrace except defects and clusters). This smooth feature is similar to the STM surface topography of the intercalated metals at the EG/SiC interface reported for In^22,23^ and Li^24^ and is distinguished from the surface topography of bare EG (mono or bilayer) and GBL in which the (6√3 × 6√3)R30° superstructure of the underlying SiC substrate is visible. The (6√3 × 6√3)R30° reconstructed structure in the GBL is formed by the 1/3 monolayer of Si adatoms on top of the SiC substrate and can easily be observed by STM images with high corrugation^21,22,25^ and in case of monolayer or bilayer EG/SiC without intercalation, this superstructure is also evidenced.^26–29^ Previous studies^22,23^ have reported the intercalation of In at the EG/SiC interface using the initially grown GBL in which the metal intercalation tends to start at the GBL areas, resulting in monoatomic and bilayer In domains. However, some parts of the initially grown sub-monolayer EG (that coexists with the GBL on the initial sample) and GBL areas are not intercalated, resulting a quite complex surface consisting of three different types of topography: (i) monolayer In, (ii) bilayer In and (iii) the un-intercalated GBL or monolayer EG. However, in our case, evidence of un-intercalated monolayer EG or GBL is not observed. This may be because of the fact that our STM is only able to probe nanosized areas, and thus misses the topographic information on the larger scale at the micron size or more, even though we have approached the STM tip on about twenty locations. Nevertheless, the dominant topographic characteristics shown in Fig. 1(a) indicate that our sample is largely intercalated by In. In more detail, as can also be seen in Fig. 1(a), the two intercalated regions are separated from each other by a step edge boundary across the image and differ in height by 1.2 Å which is shown in the height profile along the cyan line indicated in the inset of Fig. 1(a). Throughout different sample locations, we observed mainly this kind of step boundary with the same apparent height (∼1.2 Å) which varies only slightly under different imaging conditions (*i.e.* different bias voltages). Only a few step edges of larger apparent height (∼3 Å) are observed (see Fig. S2 in the ESI†). Since the step edge of the SiC (0001) is of ∼3 Å,^20,30^ and the height difference between a monolayer and bilayer EG on a SiC substrate is of 0.8 Å,^31^ we attribute the step edge boundary found in Fig. 1(a) to be formed as the intersection between the two intercalated In domains having different thicknesses on the same atomically flat SiC terrace. When further imaging this boundary step at low bias (0.1 V) as shown in Fig. 1(c) (that is enlarged from an area indicated by the green square in Fig. 1(b)) we find that the graphene honeycomb lattice is physically continuous from the low to the high regions. This indicates that both regions are covered by the graphene capping layers. It is well known from the literature that the general intercalation mechanism for metal at the EG/SiC interface occurs such that the metal atoms penetrate into the interface between the initially grown GBL or monolayer graphene and the SiC substrate at the defect sites^32^ or step edges,^24^ and break the remaining covalent bonds between the C atoms of the GBL and the Si atoms of the SiC substrate^33^ and release the GBL to form a continuous graphene sheets on top of the structure. According to this intercalation mechanism, monolayer and bilayer EG are expected to be formed as topmost layers above the intercalated In because the initially grown sample is composed of the sub-monolayer EG and GBL. In addition, defect-like structures are seen with different sizes and shapes. Notably, numerous bright triangular defects are observed only in the low intercalated region (Fig. 1(a) and (b)) which implies interesting surface characteristics. However, we later confirmed that these defects might be physical adsorbates originating from the sample preparation or from the ambient transport since they are easily removed by the tip during imaging at large tunneling current. Within the current discussion, we will not further consider them in detail.

Two-dimensional indium forms its own electronic state at the interface and modifies the electronic structure of graphene capping layers *via* doping. Here, we acquire d*I*/d*V* spectra which is proportional to the local density of states to probe the electronic structures of the system. Fig. 1(d) shows two d*I*/d*V* spectra taken in the low (blue curve) and high (red curve) intercalated regions. Around the Fermi level, both spectra reveal the linear dispersion of the π and π* bands of the graphene top layer. This again confirms that the initially grown GBL and sub-monolayer EG surface have been transformed into mono and bilayer graphene, respectively due to the intercalation since the GBL does not exhibit a linear band structure as it lacks the π states in the vicinity of the Fermi level.^25,34–36^ This also indicates that the graphene structure is essentially unperturbed and stacks with the SiC substrate as a quasi-free-standing layer mediated by the In atoms to form an EG/In/SiC heterostructure. A closer inspection around the Fermi level can be viewed in the magnified d*I*/d*V* spectra shown in Fig. 1(e) which clearly reveal two local minimums (indicated by blue and red bars) centered at ∼−0.2 eV and ∼−0.4 eV representing the Dirac points of graphene measured in the high and low regions, respectively. Through a systematic investigation, we plot a histogram of the Dirac point energy as shown in Fig. 1(f) that is measured on different locations in low and high regions resulting in an average Dirac energy shift of 0.27 ± 0.042 eV which clearly indicates a doping variation of the graphene capping layer between the two intercalated regions. The downshift of the Dirac point below the Fermi level has been observed in some intercalated metals at the EG/SiC interface such as Cu,^17,37^ Au,^5,34^ Ag,^6^ Li,^38^ and Pb^39^ which is attributed to the charge transfer from the intercalated metal to the graphene capping layer. However, the doping effect shown here indicates an interesting behavior which varies apparently due to different In thicknesses (that is ∼1.2 Å). As pointed out by Baringhaus *et al.*^40^ for Ge intercalation under EG/SiC, the graphene boundary edge between low and high intercalated regions having different carrier densities can be considered like a p–n junction. The origin of the doping difference observed in our system might be explained according to the DFT prediction^41^ which demonstrates that the n-doping of a graphene layer sitting directly above metals is increased when the metal–graphene separation reduces from 3.3 Å or less. In order to avoid any mistake in identifying the Dirac point, it is worth noting that the gap-like feature centered at ±67 meV around the Fermi level as highlighted by the shaded rectangle in Fig. 1(e) originates from a phonon-mediated inelastic tunneling in graphene.^42^ Apart from the linear band dispersion of graphene, a noticeable occupied state at ∼−1.1 eV (marked by the red triangle) in the red spectrum of Fig. 1(d) measured in the high intercalated region is observed. In contrast, we do not observe any state for the lower region which clearly indicates that this new state appears depending on the thickness of the underlying In. We also measured the d*I*/d*V* spectra in the bias voltage range between −3 V and +3 V and did not identify any other state in both regions. Since the occupied state at −1.1 eV has not been observed for the GBL or pristine graphene on SiC without intercalation,^35,39^ this state should be attributed to the electronic structure emerging from the newly formed interface. It is unclear if this occupied state arises from the main electronic contribution of the In itself as it might develop its own band structure or associated by a complicated electronic coupling between In layers and the SiC substrate or graphene at the interface. This nevertheless exhibits a very interesting electronic structure for the ultrathin In. In order to precisely describe this newly emerging state, further theoretical calculation is needed.

**Fig. 2:**
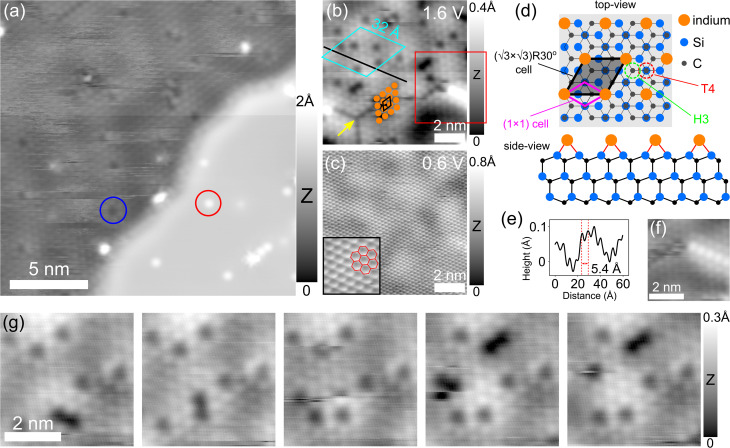
Atomic details of the low In intercalated region. (a) STM topography image (*V* = 2 V, *I* = 50 pA) of the same area indicated by the orange square in Fig. 1(a) showing atomically resolved structure for the In layer in the low intercalated region when imaging at 2 V. In contrast, on the higher layer area, no periodic structure is resolved. Blue and red circles indicate characteristic dark and bright point defects in each region. (b) Zoomed-in STM topography image (*V* = 1.6 V, *I* = 30 pA) on an area of the low region. The cyan rhombus indicates a supercell that is commensurate with (13 × 13) times the unit cell of graphene emerging from the superposition induced by the two atomic planes between the graphene capping layer and the underlying monolayer In that is also visible at this voltage. Atomic resolution of the In atoms in the low region is clearly shown; sixteen orange dots are used to highlight the triangular lattice of the In. The black rhombus indicates the In unit cell (5.4 Å). The yellow arrow points to the dark defect line as the misfit registry in the In network. The red square marks the bright out-of-plane atomic chain above the In layer. (c) Same area in (b) (*V* = 0.6 V, *I* = 30 pA) imaged at low bias voltage showing only atomic structure of the graphene top layer. Inset: STM topography image (*V* = 0.6 V, *I* = 30 pA) of an enlarged area for a better view. (d) Top and side views of the In structure in the low region with respect to the SiC substrate sketched based on the lattice parameters found in (b). The black transparent rhombus represents an In unit cell (5.4 Å) that fully matches with the (√3 × √3)R30° cell at the T4 sites of the underlying SiC. The purple rhombus represents the unit cell of the SiC (0001) substrate. (e) Height profile along the dark line in (b). (f) Zoomed-in STM topography image (*V* = 1.6 V, *I* = 50 pA) from the red square in (b). (g) Series of STM topography images acquired at 1.6 V illustrating the mobility of dark vacancies under scanning.

Height variation in the STM image is correlated with two distinct atomic phases of indium. A challenge for identification of the intercalated structures at the EG/SiC interface is that metal layers are covered by graphene preventing direct access to the confined atoms underneath. In the STM technique, the tip is sensitive to the density of states located several atomic layers below a surface that has been used to directly identify the buried atomic structures, for example, Si vacancies and domain boundaries in yttrium silicide surface,^43^ (7 × 7) reconstruction of Si (111) below monolayer graphene^44^ and SiC interface structure below monolayer graphene.^45,46^ Accordingly, when the two atomically indistinguishable surface regions shown in Fig. 1(b) and (c) obtained at 1 V and 0.1 V are imaged at 2 V, they differ significantly in their atomic contrasts (see Fig. 2(a)). To our surprise, a new atomic structure consisting of a well-ordered arrangement of tiny dots is clearly seen on the lower domain (left part of the image) with a surface corrugation of ∼0.25 Å at this bias voltage. In contrast, the higher surface domain appears as a highly flat terrace without any periodic structure (with a corrugation of ∼0.02 Å, lower right part). At this bias voltage, both regions are also composed of dark and white defects as indicated by blue and red circles. This observation immediately reflects that the intercalated surface is composed of two different atomic phases with distinct structures.

Lower intercalated regions exhibit an ordered indium atomic array that epitaxially matches the (√3 × √3)R30° cell of the underlying SiC. The details of the low region are further shown in Fig. 2(b). The high resolution STM image obtained at 1.6 V from a zoomed-in low area reveals an array of bright dots with an average diameter of ∼3.1 Å which are attributed to individual In atoms below graphene. These atoms are arranged in a triangular lattice structure resembling a face-centered cubic (fcc) (111) plane with an atom-to-atom distance of ∼5.4 Å (as shown in Fig. 2(e) obtained from the black line indicated in Fig. 2(b)) reflecting the size of its unit cell as indicated by the black rhombus above the sixteen orange dots used to highlight the atomic arrangement in the low layer. In contrast, when imaging this area at 0.6 V or at a lower bias voltage, only atomic resolution of the graphene honeycomb lattice is displayed as can be seen in the STM image of Fig. 2(c). This clearly demonstrates that the In atoms form a highly crystalline thin layer below the graphene capping layer as the atomic structure of graphene is clearly resolved at a lower bias voltage close to the Fermi energy.^26^ We image this area at different bias voltages and observe that the atomic structure of the 2D In is only visible in the bias range between 1.5 V and 2.2 V. At larger negative and positive voltages, the STM images appear very unstable without special features. Previous studies^3,5,6,14–17^ have demonstrated various metal elements intercalated at the EG/SiC interface in which the first intercalated metal layer tends to match (1 × 1) epitaxially to the SiC substrate such that each metal atom is bound directly to the topmost Si atom of the SiC substrate. The unit cell of 6H-SiC (0001) is known to be 3.08 Å^46,47^ which is considerably smaller than that of the intercalated In (5.4 Å) found here. Thus, such a (1 × 1) match cannot be considered for this In network in the low region. Indeed, we found that the In unit cell (5.4 Å) coincides with the (√3 × √3)R30° cell of the underlying SiC (that is √3 × 3.08 = 5.33 Å). Therefore, in order to match the two lattice planes, each In atom must bind to either Si atom, carbon atom (T4 site) or hollow (H3 site) of the SiC topmost layer. Among these sites, the T4 site is proved to be energetically more favorable than the H3 and the hollow sites in various epitaxial 2D systems formed on the SiC (0001), for example, in case of Si adatoms chemisorbed and formed the (√3 × √3)R30° reconstruction on the SiC surface^48,49^ and recent observations for In intercalated at the EG/SiC interface.^22,23^ Notably, the atomic characteristics of the intercalated In atoms in our case are very similar to that of Sn atoms adsorbed on the SiC (0001) substrate without the graphene capping layer.^50,51^ In this system, each Sn atom is found located at the T4 positions and energetically bound to its three adjacent Si atoms on the SiC substrate. Such observations lead us to conclude that the well-ordered In structure found in the low intercalated region is commensurate strictly with the (√3 × √3)R30° superlattice at T4 positions on the SiC substrate as illustrated in Fig. 2(d) where the (√3 × √3)R30° cell is indicated by the black transparent rhombus (the SiC unit cell is also indicated as a purple rhombus). Considering the (1 × 1) atomic matching commonly occurring in various 2D metals at the EG/SiC interface as mentioned above, and the In triangular network on the SiC (0001) substrate without the capping graphene layer,^18^ it is still unclear under which circumstances the (√3 × √3)R30° triangular network of In is formed. Nevertheless, the ordered structure of ultrathin In provides strong evidence that the intercalated 2D In significantly interacts with the SiC substrate within the interface. In turn, the topmost atomic layers of the SiC substrate can be considered as a considerably important epitaxial template for 2D metal to be formed. It is also noted that the In lattice in this region has the same crystallographic orientations with the graphene top layer which implies that graphene coverlayer is only lifted up due to the intercalation and maintains its registry with the underlying SiC substrate. This behavior is consistent with the previous DFT calculation^3^ which revealed that adding the graphene to the intercalated In/SiC only affects the band filling without modifying the stability of the intercalated metal.

Coexisting with the atomic structure of the In layer, a large superstructure arising as a result of the lattice mismatch between the monolayer graphene and the underlying In lattice is visible as faint corrugation lines indicated by a cyan rhombus in Fig. 2(b) and can also be clearly seen in Fig. 2(g). At first glance, this superstructure may be equivalent to the well-known (6√3 × 6√3)R30° cell of the reconstructed SiC (0001) because it has a size of (13 × 13) times the graphene unit cell (that is, 13 × 2.45 ≈ 32 Å).^21,27,29,52^ However, since the graphene layer is lifted and becomes a quasi-free-standing layer due to the In intercalation, this supercell should have a different origin. In more detail, Forti *et al.*^17^ have demonstrated in case of Cu intercalated at the EG/SiC interface that the Cu atoms in a monolayer match epitaxially with the SiC substrate such that a (2 × 2) Cu cell (lattice constant of 2.66 Å) is commensurate with the (√3 × √3)R30° cell of the SiC substrate. In such an atomic arrangement, the (13 × 13) supercell emerges from the superposition induced by the two atomic planes between monolayer graphene and monolayer 2D metal. Within this cell, the underlying metal atoms register with the graphene network either directly below the hollow center of the graphene hexagon or below the A or B sublattice atom resulting in a superlattice varying potential. Since the In intercalated layer matches in a (1 × 1) ratio with the (√3 × √3)R30° superlattice of the SiC substrate (the In unit cell of 5.4 Å is almost twice the size of the above-mentioned Cu unit cell), the potential modulation periodicity induced by the atomic registry between In and graphene is similar to that between Cu and graphene. In addition, this superstructure is also present in other intercalated metal systems at the EG/SiC interface such as Au^5,14^ and Cu^37^ showing consistency with our observation.

A range of defect types exists in the low intercalated layer, enabling visualization of the ultrathin nature of 2D In. As can be seen in the STM images of Fig. 2(a) and (b), different types of defects including dark point defects (indicated by a blue circle), dark defect line (yellow arrow) and bright atomic chain (red square) are clearly visible. First, the dark point defects are found to be located precisely at the lattice sites of In atoms that can be attributed to the indium vacancies (missing atoms) in the In layer. Under repeated scanning as displayed in the series of STM images acquired at 1.6 V shown in Fig. 2(g), these vacancies can be seen displacing randomly inside the scanning area. The observation of the hopping vacancies indicates that the In atoms in the lattice are vulnerable under tip scanning due to the ultrathin nature of the In, however, the atomic network remains unaltered exhibiting its strong interaction with the well-defined structured SiC substrate. Second, the fragile nature of the intercalated In is also exhibited by the observation of the dark defect line marked by a yellow arrow in Fig. 2(b) which is formed and disappears upon repeated scanning. This dark defect line can be explained in a way that the (√3 × √3)R30° In lattice is laterally shifted by one Si atom to the next nearest lattice site induced by the perturbation of the tip-induced electric field creating different atomic domains, separated by a misfit line in the In atomic plane.^50^ Third, a bright atomic-like chain is also observed in some areas as indicated by the red square in Fig. 2(b) and its details are shown in Fig. 2(f). This structure is formed out-of-plane above the In layer below graphene in the low region and stacks with the underlying In layer similarly to an AB stacking. As evidenced, these bright atoms have nearly the same diameter and the atom-to-atom distance (which is measured to be 5.3 Å) as the lower In layer. In strong contrast to the dark vacancies, these atomic chains are stable under repeated scanning. In our understanding, this atomic chain might be evidence of a starting nucleation growth for a second In layer.

**Fig. 3:**
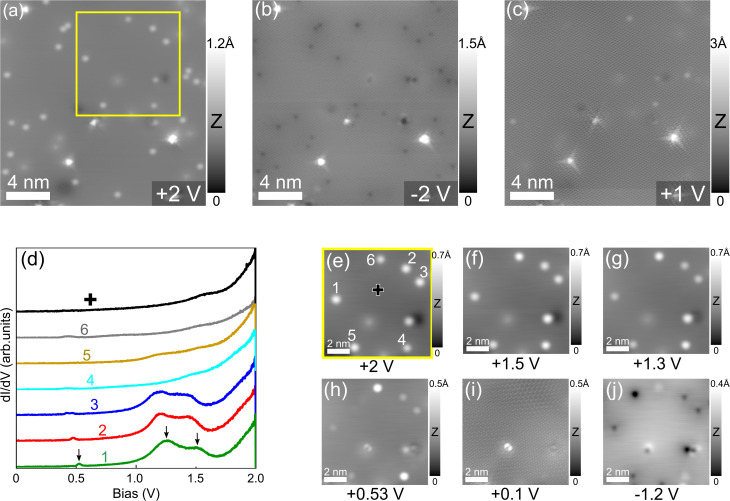
Surface characterization of the high intercalated region. (a–c) STM topography image of the same area in the high intercalated region at different bias voltages: 2 V, −2 V and 1 V, respectively (tunneling current: *I* = 100 pA). The atomic resolution of In atoms is not resolved here, only intrinsic point defects are visible as bright protrusions, dark cavities at different bias voltages. Triangular defects belong to the graphene coverlayer. (d) d*I*/d*V* spectra measured on six defects marked from 1 to 6 indicated in (e). The black spectrum is probed on a clean surface region indicated by a black cross in (e). (e–j) Bias-dependent STM topography images obtained from the area indicated as a yellow square in (a).

Bias-dependent imaging is unable to verify the structure of “thick” indium intercalated regions which is hypothesized to be composed of more than one metallic layer preventing individual atoms to be resolved. In Fig. 3(a) we show a zoomed-in STM image obtained from a smaller area which appears highly flat at 2 V. As pointed out above, this region is about 1.2 Å higher than the lower intercalated region suggesting that it may be composed of more than one In layer. This assumption will be further clarified in the later discussion. However, at this stage, we speculate that when more than one monoatomic metal layer with an ordered structure is stacked on top of each other such as in an AB or ABC stacking, the strong electronic coupling induced by the different atomic planes of identical density will prevent individual atoms in the topmost metal layer to be resolved. This makes the atomic structure identification for this intercalated region challenging. Apart from this aspect, different point defects are clearly imaged in this region because they have a considerably different electronic density as compared to that of the In atoms. As clearly shown also in Fig. 3(a) the bright point defects on the high intercalated region are visible at 2 V as round protrusions. These defects exhibit a strong bias-dependent contrast. When the bias polarity is switched from 2 V to −2 V, they appear as dark cavities as can be seen in Fig. 3(b). The positions of these bright protrusions are fully commensurate with the dark cavities except a few bright triangular defects. The bias-dependent contrast of the defects shown here is somewhat in agreement with the previous observation of vacancies in a bilayer In below graphene.^22^ At this stage of analysis, we tentatively attribute these point defects to monovacancies of the intercalated In layers. However, in order to precisely conclude that these are vacancies in the In layer, further investigation should be considered. In addition, it is worth noting that the bright triangular defects coexisting with the atomic resolution of graphene are clearly displayed at 1 V or lower bias voltages as shown in Fig. 3(c), indicating that they instead originate from the graphene atomic plane. Indeed, triangular defects in graphene have been well observed by STM images^53,54^ which results from local charge transfer between the defect and the three adjacent carbon atoms in the graphene lattice.^55–57^ These defects are attributed to be either monovacancies or foreign atoms substituted monovacancies in the graphene lattice.^55^

Differential (d*I*/d*V*) spectroscopy is further used to elucidate the electronic signatures of the defects in 2D indium. Fig. 3(d) presents different d*I*/d*V* spectra obtained with the tip placed above six defects marked from 1 to 6 in Fig. 3(e) which is an enlarged area from the yellow square in Fig. 3(a). The spectra taken above these defects mainly exhibit three unoccupied states as indicated by black arrows, while the reference spectrum (black curve) taken at a clean surface area marked with a black cross does not show any pronounced resonance in this energy range. In addition, no other electronic states are probed in the energy below the Fermi level. As can be seen in detail, the position of these occupied states varies slightly over different defects suggesting that they are not all identical. This is further confirmed in the series of STM topography images shown in Fig. 3(e)–(j) taken at the bias voltages corresponding to the resonance peaks from the d*I*/d*V* spectra. As clearly seen, the defects vary slightly in sizes and contrast. This implies that they do not sit on the equivalent lattice sites of the topmost In layer if one assumes that this layer is formed with a periodic structure. The d*I*/d*V* spectra taken on the defect 4 and 6 do not show any pronounced peak and have different contrasts in the STM images, notably those shown in Fig. 3(h) and (i). This suggests that they may reside in the deeper atomic planes and have a different origin such as Si adatoms from the SiC substrate.

### 3.2 Tip-induced surface manipulation

**Fig. 4:**
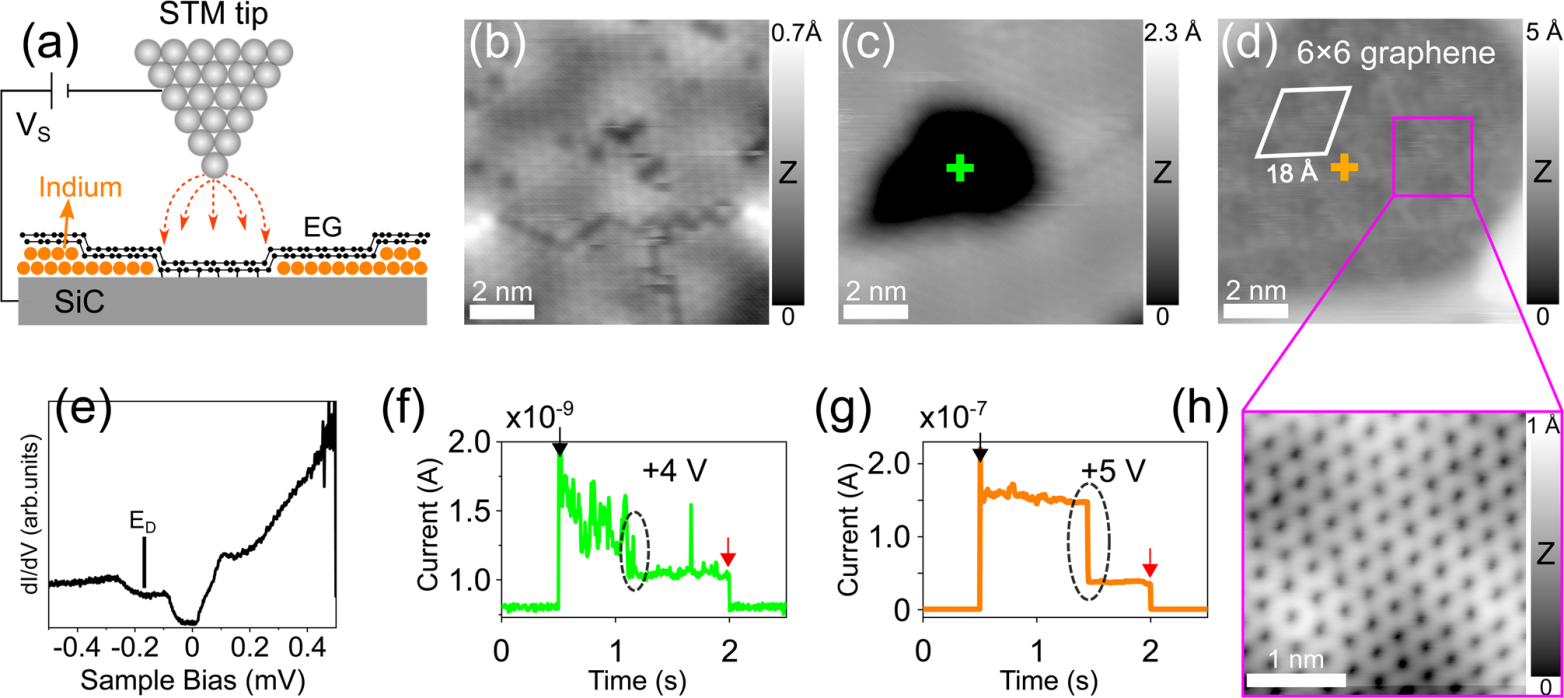
Tip-induced manipulation of intercalated In below EG on SiC. (a) Sketching of STM tip manipulation to de-intercalate the In atoms below graphene by applying a bias voltage pulse between the tip and the sample. (b) Initial STM topography image (*V* = 2 V, *I* = 30 pA) of a low intercalated area already shown in Fig. 2(b) as a starting area for manipulation. (c) STM topography image (*V* = 1.6 V, *I* = 30 pA) of the same location after the first voltage pulse of 4 V is applied. (d) STM image (*V* = −1.5 V, *I* = 30 pA) of the same location after the second pulse of 5 V is further applied. The green and orange crosses indicate the tip positions. The superstructure with a periodicity of 18 Å (white rhombus) typical for a 6 × 6 reconstruction of SiC is clearly observed indicating that only EG (referred to as 6 × 6 graphene) sits on top of SiC without intercalated In atoms. (e) Typical d*I*/d*V* spectrum obtained on the deintercalated area showing the characteristic density of states of EG on SiC; *E*_D_ marks the position of Dirac energy (at ∼−175 mV) shifted below the Fermi level. (f and g) Tunneling current traces during the tip voltage pulses inducing surface modification as displayed in (c) and (d). The black and red arrows indicate the current switching from the initial imaging conditions (*V* = 2 V, *I* = 30 pA) to the bias voltage pulses. The current drop marked by the dashed ellipses indicates the tip–sample distance increase when the intercalated In atoms migrate away from the tip location. (h) Atomic resolution STM image (*V* = 0.02 V, *I* = 100 pA) of a 6 × 6 graphene area marked as a purple square in (d) showing the atomic honeycomb lattice.

Tip-induced surface manipulation enables direct visualization and engineering of In layers at the EG/SiC interface (as described in Fig. 4(a)). Fig. 4(b) shows the initial area (which is the same area in Fig. 2(b) of the low intercalated region) where we placed the STM tip at a fixed position above the surface. Two positive bias voltage pulses of 4 V and 5 V were consecutively applied. The tip positions are indicated as green and orange crosses in Fig. 4(c) and (d), respectively. The details of the tunneling process between the tip and the sample are shown in the current traces presented in Fig. 4(f) and (g) in which the current drop (circled by the dashed ellipses) indicates the surface modification induced by the voltage pulses. This is because when the In atoms are removed at the location below the tip, the tip–sample distance increases, thus reducing exponentially the tunneling current. Note that the first and the last steps in the current traces (indicated by black and red arrows) are indications of the tip switching between the initial imaging condition (*V* = 1.6 V, *I* = 30 pA) and the applied bias voltages (4 V and 5 V). As a result, after the first pulse of 4 V, the first dark hole with a size of ∼5 nm and a depth of ∼1.4 Å is formed as can clearly be seen in the STM image in Fig. 4(c). This hole is further extended to a larger diameter of ∼10 nm with a depth of ∼2 Å after a second 5 V pulse was applied (Fig. 4(d)). The area inside the hole now appears as a highly corrugated surface with a periodicity of 18 Å (indicated by a white rhombus). The magnified STM image from this area at low bias voltage (0.02 V) shown in Fig. 4(h) reveals the atomic resolution of the graphene structure. Importantly, the periodicity of 18 Å corresponds to a 6 × 6 periodicity (the unit cell of SiC is 3.08 Å) which is a typical moiré pattern belonging to the 6√3 × 6√3 reconstruction of the underlying SiC.^25,35,45,58^ This is an important indication that the area inside the hole is composed of graphene sitting directly above the 6√3 × 6√3 reconstructed SiC substrate without the intercalated metal (hereafter denoted as 6 × 6 graphene). We refer to this observation as the “de-intercalation” of the underlying In induced by the tip voltage pulse. This also reflects that following the de-intercalation, the topmost EG layer remains intact and stacks directly with the SiC substrate followed by the restoration of the underlying GBL. However, to understand the exact mechanism of how the GBL is recovered under the top EG layer, further investigation is required.

**Fig. 5:**
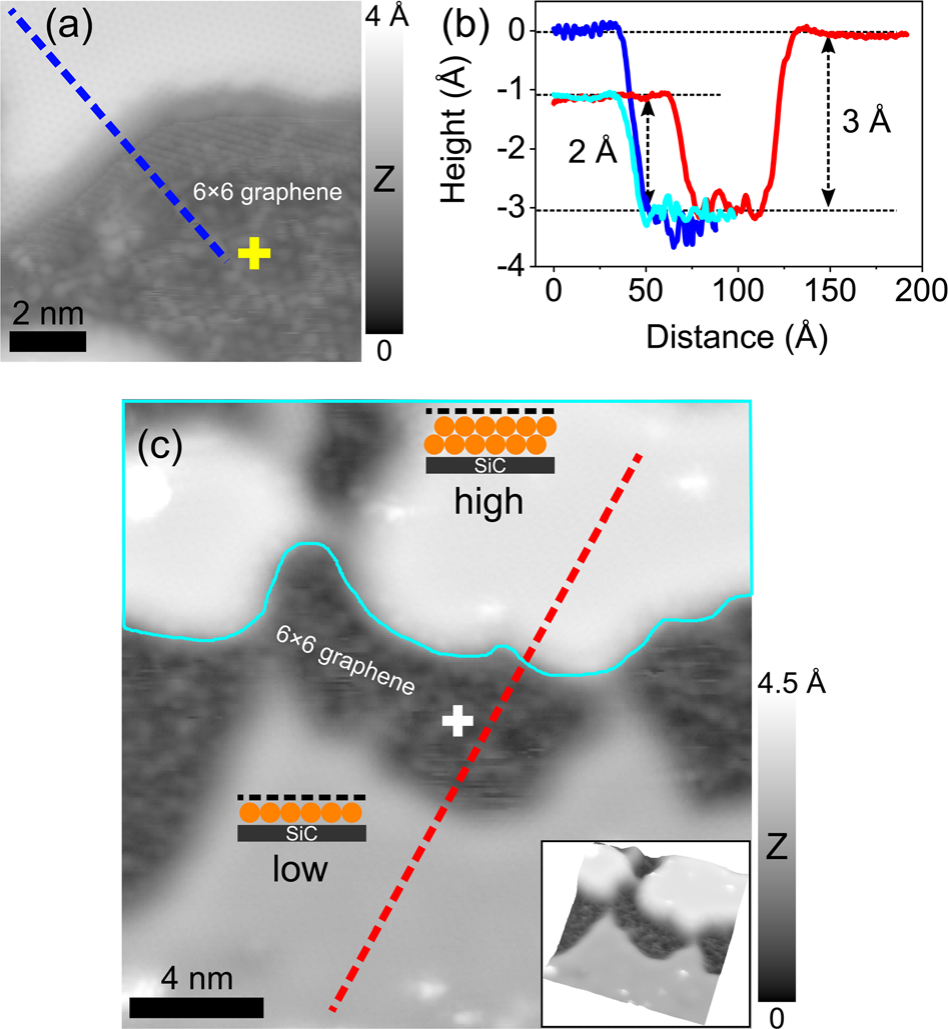
Tip-induced engineering of intercalated 2D metal. (a) STM topography image (*V* = −1 V, *I* = 100 pA) of a high intercalated area after applying a voltage pulse of 5 V (yellow cross indicates the tip position) showing an atomic step between the intercalated and de-intercalated (6 × 6 graphene) regions. (b) Height profiles along the blue and red curves indicated in (a) and (c), respectively. The cyan curve is the height profile measured across the step edge between the low intercalated and the de-intercalated region shown in Fig. 4(d) (the line is not shown due to small image size). (c) STM topography image (*V* = 1 V, 10 pA) of a large area in a low intercalated region after a voltage pulse of 8 V was applied (white cross) revealing newly formed bilayer In (cyan bordered, upper part) with brighter topographic contrast, de-intercalated (black regions) and untouched lower intercalated region. The inset shows the three-dimensional image of the area for better visualization.

The modification of intercalated indium suggests the alteration of the local electronic structure. We acquired d*I*/d*V* spectra at the de-intercalated area to investigate how the electronic structure of the heterostructure is modified. As shown in Fig. 4(e), the spectrum exhibits the electronic structure of graphene with the Dirac point centered at −175 mV below the Fermi level, further strengthening our conclusion for the presence of graphene in this area which is typically n-doped induced by a direct charge transfer from the SiC substrate without mediating metal atoms.^59,60^ Note that the Dirac point shift observed in our case is slightly different as compared to the reported energy shift of the typical EG/SiC system (which is about −450 mV below the Fermi level^60^). This is because in our case the newly formed pristine graphene area is very small after the voltage pulse, the surrounding area is still intercalated by In atoms, and thus, we expect a different electronic coupling between graphene and the SiC substrate. We again exclude that this de-intercalated area is composed of the GBL because in this case, no linear band dispersion typical for graphene should be observed.^45^ Note that the Dirac point shift in this spectrum is irrelevant with the shift induced by In atoms in the low and high intercalated regions that has been discussed in Fig. 1(d)–(f). Next, we also applied this tip-induced manipulation in the higher intercalated region which is presented in the STM image of Fig. 5(a). Similarly, the reconstructed 6 × 6 periodicity associated with graphene hexagonal atomic structure is again observed in the de-intercalated area (lower part of the image). Based on this observation, we conclude that the voltage pulse has successfully redistributed the intercalated material under the graphene capping layer in the sense that the In atoms are compressed and shifted laterally to the nearby region outside the hole. Outside of the de-intercalated region, we have observed two types of surface topography after the voltage pulse. (i) The surface area in the proximity area around the hole remains unaltered which can be observed in Fig. 4(c) and 5(a). The d*I*/d*V* spectra measured on such locations on both low and high intercalated regions (see Fig. S3, ESI†) consistently show similar electronic structures to those presented in Fig. 1(d) (red and blue curves). At this point, it is apparent that In atoms have been shifted away from the de-intercalated area around the tip. However, they are still strongly bound to the topmost Si atoms of the SiC substrate to maintain the ordered structures. (ii) Strikingly, in some cases, under a larger voltage pulse ranging between 6 V and 8 V, the nearby areas around the de-intercalated area in the low intercalated regions appear to have a higher apparent height implying that the intercalated material in these regions has been reconstructed to form a new structure. This will be further discussed in detail below.

The tip-induced modification mechanism can be explained *via* vibrational excitations that induce bond breaking within the metal layer through inelastic electron tunneling induced by the STM tip.^61–63^ In our measurement, as the graphene still covers the top of the structure, it indicates that the tunneling current has passed through the EG layer and interacted with the underlying In. We also found that the surface modification can be achieved using both positive and negative voltages, however, the positive bias voltage performs with a better control. It should be noted that de-intercalation of metal confined at the EG/SiC interface is normally achieved *via* sample annealing at elevated temperature above 800 °C (ref. 64 and 65) followed by the restoration of the BGL. The tip-induced manipulations of intercalated non-metallic materials were previously demonstrated to de-intercalate, for instance, water molecules trapped between graphene and a mica surface.^66^ To the best of our knowledge, the controlled de-intercalation of metal at the EG/SiC interface through tip-induced manipulation has not previously been established so far.

Tip-induced interface manipulation is a powerful method to directly visualize and investigate the properties of 2D metals below the graphene capping layer. Upon de-intercalation, the metal thicknesses in the low and high intercalated regions can easily be explored by measuring the height of the atomic steps between the intercalated and de-intercalated (6 × 6 graphene) regions. As can be seen in Fig. 5(b), the blue and cyan curves show the height profiles crossing the step edges shown in Fig. 5(a) and 4(d), respectively (the line crossing step edge in Fig. 4(d) is not drawn in the image due to its small size). Accordingly, the thickness of the higher domain is measured to be ∼3 Å (blue curve) while that of the lower one is ∼2 Å (cyan curve). We have systematically investigated the intercalation heights of low and high regions by performing the tip voltage pulse over different locations and obtained average heights of 2.13 ± 0.25 Å and 3.38 ± 0.33 Å, respectively. The thickness of 3.38 ± 0.33 Å is attributed to the height of an In bilayer under graphene/SiC as previously demonstrated.^22^ Note that this value is also close to the thickness of the Si topmost bilayer on SiC (0001).^20,30^ Thus, we now attribute the higher intercalated region to be composed of an In bi-atomic layer and infer the lower intercalated region as a monoatomic layer. Indeed, first-principles DFT calculations from Orimoto *et al.*^32^ pointed out that upon intercalation of a Cu monolayer at the GBL/SiC interface, the graphene capping layer is lifted by 2 Å which apparently supports our observation. The thickness of the In monolayer (2.13 ± 0.25 Å) in the low regions is higher than half that of the bilayer (high) region (that is 3.38 : 2 = 1.69 Å). This suggests interesting heterostructural strain-induced behaviors in both low and high intercalated regions. As demonstrated in previous STEM and DFT studies,^7^ the bonding characters in an EG/metal/SiC heterostructure for bilayer Ga and In evolves from covalent at the SiC/metal interface (with interspacing between SiC and the first Ga layer of 2.19 Å) to metallic between the first Ga layer and the second Ga layer (with a larger interspacing of 2.36 Å) and to nonbonded vdW-like at metal/graphene interface. Assuming that our first and second In layers stack with the SiC substrate and EG under similar conditions, one could expect that the apparent height of the bilayer region here must have a thickness at least larger than two times that of the low regions (that is 4.26 Å). Since the thickness of the In bilayer region is measured to be 3.38 Å, it suggests that the interaction of the interfacial In layers with graphene capping layers in the high region is stronger than that in the monolayer (low) region.

Apart from the de-intercalation ability of metal layers below graphene, the tip-induced manipulation demonstrated here is a versatile tool to control the number of intercalated layers with atomic precision, through which the remarkable dynamic behavior of the interfacial In atoms is revealed. As already mentioned above, upon the tip voltage pulses, some areas around the tip location in the low intercalated region appear with a higher apparent height. In Fig. 5(c), we show the STM image in a low intercalated area after a voltage pulse of 8 V is applied (tip position is indicated by the white cross). Strikingly, the upper parts in the image appear with brighter contrast implying a higher apparent height (indicated by “high”, contoured with cyan in the upper part). The corresponding three-dimensional image is shown in the inset for a better visualization. The height profile crossing different terraces shown in Fig. 5(b) (red curve) reveals that the newly formed region is measured to be ∼3 Å which has the same height as that measured on the initial high intercalated region as indicated in Fig. 5(a) (blue curve). This surprising observation implies that the voltage pulse has modified the initial In monoatomic layer in the low intercalated region and converted it into a bi-atomic layer structure (see the atomic structures in the inset laid on each part of the image in Fig. 5(c)). This newly formed bilayer structure is usually formed under a reasonably large voltage pulse between 6 V and 8 V. The mechanism behind this layer conversion can be understood in a way that the In atoms are thermodynamically excited by the tunneling current induced by the STM tip to a high energy state that overcomes the energy barrier formation of a monoatomic In layer to reconstruct a bilayer with more stability. Throughout various tip voltage pulses carried out at different locations in the low regions, only the In bilayer is found while trilayer or higher layer order has never been observed. Previous DFT prediction^3^ suggests that the strong metal–SiC covalent bonds play an essential role in providing a strong thermodynamic driving force for an In bilayer to be formed as the most stable atomic phase at the EG/SiC interface.

This finding is reinforced by the fact that when we carried out tip voltage pulse in the high intercalated region, the 2D In is only de-intercalated. No newly formed structures with a thickness other than a bilayer are found. In fact, we found that the monolayer In is very easily perturbed by changes in the tip conditions such as applied bias voltages, bias polarity or tunneling current while the bilayer region appears more stable. Therefore, the tip-induced manipulation not only de-intercalates the In material but can also be used as a versatile tool to engineer the intercalated layers at the EG/SiC interface.

## 4. Conclusions

In conclusions, this work presents an experimental demonstration of the detailed structures of 2D indium stabilized at the EG/SiC interface using cryogenic STM. Two atomic phases corresponding to monoatomic and bilayer In structures stabilized under graphene are revealed. On the In monolayer, a triangular lattice matching epitaxially with the (√3 × √3)R30° periodicity of the underlying SiC substrate is identified. In contrast, no clear indication of the atomic arrangement is probed for the In bilayer. This observation clearly demonstrates the significant importance of the SiC substrate as an epitaxial template for 2D crystalline In to be formed. We have successfully used tip-induced manipulation as a versatile tool to either de-intercalate or reconstruct the intercalated materials while the graphene top layer remains unaltered. This allows microscopic insight into the ultrathin In at the EG/SiC interface with respect to intercalation thickness, good control over the number of metal layers and thermodynamic stability of In at the interface. Our results contribute to exploring the atomic and electronic structure of the confined metal, strongly emphasizing on tip-induced manipulation as a first demonstration for the intercalated metal at the EG/SiC interface.

## Author contributions

V. D. Pham designed, performed all the STM experiments, analysed the data and wrote the manuscript. J. A. Robinson and C. Dong designed and prepared the intercalation samples. All authors contributed critical revisions and gave final approval of the manuscript.

## Conflicts of interest

There are no conflicts to declare.

## Supplementary Material

NA-005-D3NA00630A-s001
