# Supplementary material for: Atomic structures and interfacial engineering of ultrathin indium intercalated between graphene and a SiC substrate
Source: Nanoscale Adv. 2023 Sep 8;5(20):5601–12. doi: 10.1039/d3na00630a (PMC10563832; doi:10.1039/d3na00630a)
Supplement: NA-005-D3NA00630A-s001 [file NA-005-D3NA00630A-s001.pdf]

Electronic Supplementary Material (ESI) for Nanoscale Advances.
This journal is © The Royal Society of Chemistry 2023

Supplementary Information: *Atomic structures and interfacial engineering of ultrathin indium intercalated between graphene and SiC substrate*

Van Dong Pham,*[a] Chengye Dong [e,f] and Joshua A. Robinson [b,c,d,e,f,g,h,i]

[a] *Paul-Drude-Institut für Festkörperelektronik, Leibniz-Institut im Forschungsverbund Berlin e.V., Hausvogteiplatz 5–7, 10117 Berlin, Germany.*

[b] *Department of Materials Science and Engineering, The Pennsylvania State University, University Park, PA, USA.*

[c] *Center for Nanoscale Science, The Pennsylvania State University, University Park, PA, USA.*

[d] *Department of Physics, The Pennsylvania State University, University Park, PA, USA.*

[e] *2-Dimensional Crystal Consortium, The Pennsylvania State University, University Park, PA, USA.*

[f] *Center for 2-Dimensional and Layered Materials, The Pennsylvania State University, University Park, PA, USA.*

[g] *Materials Research Institute, The Pennsylvania State University, University Park, PA, USA.*

[h] *Department of Chemistry, The Pennsylvania State University, University Park, PA, USA.*

[i] *Center for Atomically Thin Multifunctional Coatings, The Pennsylvania State University, University Park, PA, USA.*

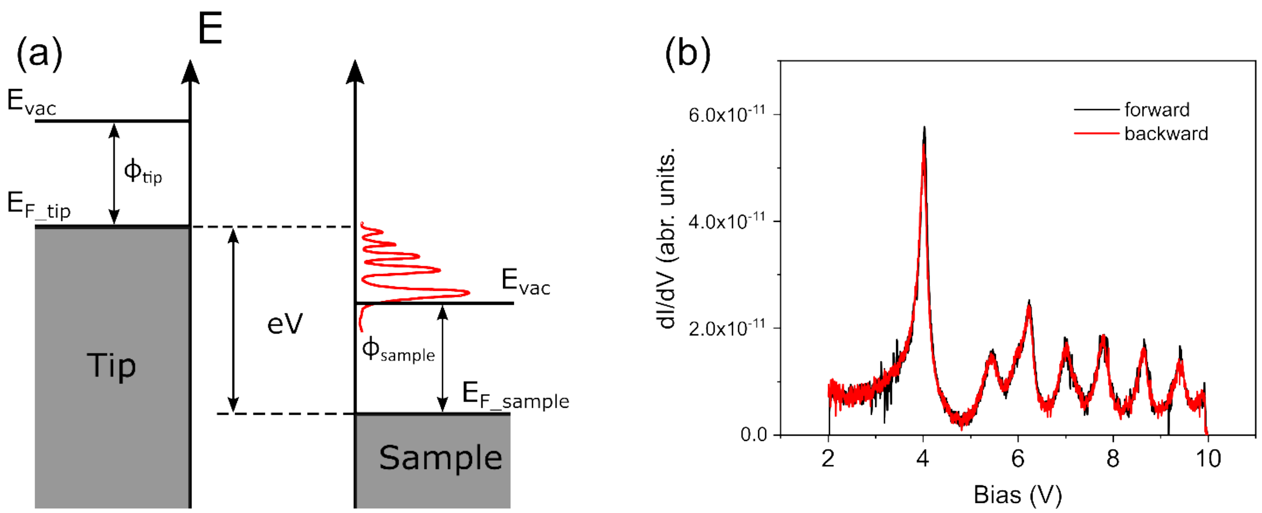

Fig. S. 1. Calibration of STM tip using field emission regime (FER). (a) Schematic illustration of a STM junction at high bias voltage in FER regime yielding hydrogen-like resonances (red) confined in the vacuum region between the tip and sample. (b) FER spectra (dI/dV spectra with closed feedback loop) obtained on clean graphene/SiC showing series of resonance peaks.

To calibrate the tip (in situ) without changing the samples, we perform conductance spectroscopy in the field emission regime (FER) by applying high positive bias voltage between the tip and the sample (typically

between 2 V and 10 V). In the FER regime, the Fermi level of the tip is higher than the vacuum level of the sample. Thus, the electric field induced by the tip lifts up the potential barrier above the vacuum level of the sample, giving rise to a set of hydrogen-like states confined to the vacuum region between the tip and the sample. We repeatedly record two dI/dV spectra by ramping the bias forward from 2 V to 10 V and and backward from 10 V to 2 V until two series of pronounced peaks in both voltage bias directions are well-observed with sharp resonance peaks without instability. We believe that the high electric field emitted from the tip helps somehow "re-form" the tip apex and remove the non-metallic dirt attached to the tip. This technique has been applied during our work and shown very high fidelity.

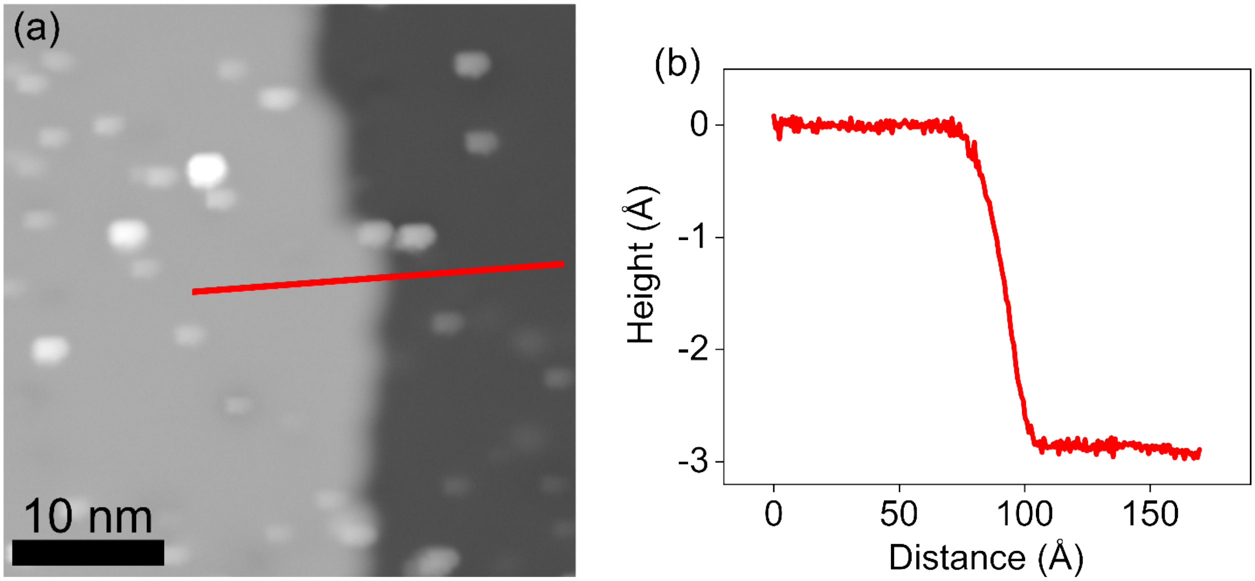

Fig. S. 2. (a) A SiC step edge with the height of 3 Å as shown in the height profile in (b).

We observe few step edges in our sample with the height profile of 3 Å. This step height is attributed to the original SiC step edge, however both regions shown here are also fully intercalated by indium (exhibiting smooth surface feature). This step edge is distinguished from the step edge shown in Fig. 1 (a) with the height of only ~ 1.2 Å which is formed due to different indium coverage on the same atomically flat SiC terrace.

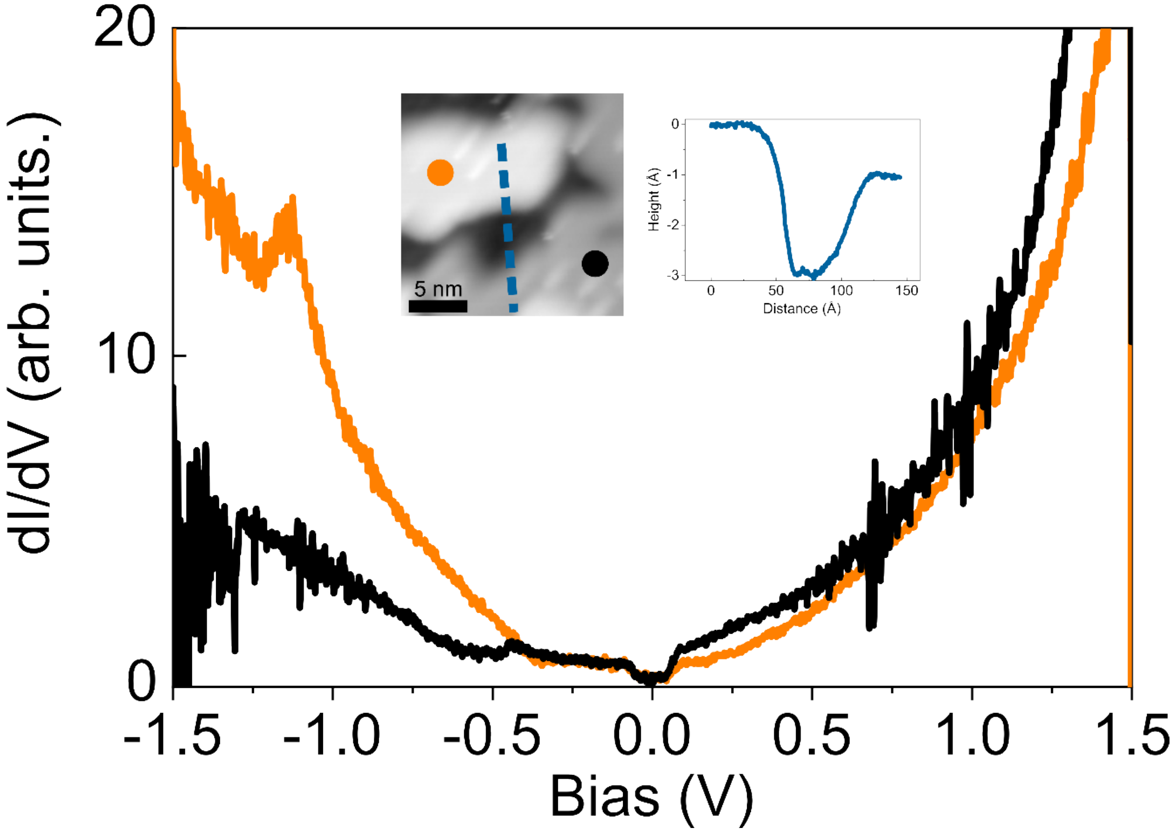

Fig. S. 3. dI/dV spectra taken on a low intercalated region after tip-induced manipulation is applied. The left panel of the inset shows STM image of the location after a voltage pulse of 6 V is used. The brighter region is formed due to this pulse. The right panel shows the heigh profile along the navy line in the right panel.

In Fig. S. 3, the orange spectrum is taken on the newly formed higher region (brighter contrast, marked as orange dot in the STM image of the inset) which is formed after the voltage pulse. The black spectrum is taken on the lower region (marked as black dot in the inset). Both spectra show very similar features with initial spectra taken on the low and high regions in Fig. 1 (d). This indicates that upon tip manipulation, the newly formed region is identified as bilayer indium showing exactly the same electronic structures with that of the initial high intercalated region.
